# Breast cancer and the steadily increasing maternal age: are they colliding?

**DOI:** 10.1186/s12905-024-03138-4

**Published:** 2024-05-14

**Authors:** Ambrogio P. Londero, Serena Bertozzi, Anjeza Xholli, Carla Cedolini, Angelo Cagnacci

**Affiliations:** 1 Department of Neuroscience, Rehabilitation, Ophthalmology, Genetics, Maternal and Infant Health, University of Genoa, Largo Rosanna Benzi, 10, Genoa, 16132 Italy; 2 Obstetrics and Gynecology Unit, IRCCS Istituto Giannina Gaslini, Via Gerolamo Gaslini, 5, Genoa, 16147 Italy; 3 Breast Unit, University Hospital of Udine, Udine, 33100 Italy; 4 Ennergi Research (Non-Profit Organisation), Lestizza, 33050 Italy; 5 Academic Unit of Obstetrics and Gynecology, IRCCS Ospedale San Martino, Genoa, 16132 Italy

**Keywords:** Breast cancer, Young women, Advanced maternal age, Delayed childbearing, Breast cancer screening

## Abstract

**Background:**

Pregnancy-related cancers are mostly breast cancers, and their incidence is likely to increase as a result of the modern trend of delaying childbearing. In particular, advanced maternal age increases breast cancer risk, and younger breast cancer patients are more likely to die and metastasize. This study compared a population with a high incidence of delayed childbearing with another population with a lower mean age at childbirth in order to determine whether breast cancer diagnosis and childbearing age overlap.

**Methods:**

We retrospectively analyzed multiple data sources. The Surveillance, Epidemiology, and End Results (SEER) program, the United States National Center for Health Statistics as part of the National Vital Statistics System, the United Nations Population Division, the GLOBOCAN Cancer Observatory, the CLIO-INFRA project database, the Human Fertility Database, and anonymized local data were used.

**Results:**

As women’s age at delivery increased, the convergence between their age distribution at breast cancer diagnosis and childbearing increased. In addition, the overlap between the two age distributions increased by more than 200% as the average age at delivery increased from 27 to 35 years.

**Conclusions:**

As women’s average childbearing age has progressively risen, pregnancy and breast cancer age distributions have significantly overlapped. This finding emphasizes the need for increased awareness and educational efforts to inform women about the potential consequences of delayed childbearing. By providing comprehensive information and support, women can make more informed decisions about their reproductive health and cancer prevention strategies.

## Highlights

• As the age of women at childbirth increased, the convergence of their age distribution at breast cancer diagnosis grew.

• When the average age at delivery increases from 27 to 35 years, the overlap between age distribution at delivery and that at breast cancer diagnosis increases by more than 200%.

• These results highlights the need for more education and awareness about the risks of delayed childbearing. Women can make better reproductive health and cancer prevention decisions with comprehensive information and support.

## Condensation

As women’s average childbearing age has progressively risen, pregnancy and breast cancer age distributions have overlapped. While the average delivery age has climbed from 27 to 35 years in the last three decades, the overlap between breast cancer diagnosis and delivery age distributions has increased by almost 200%. This finding emphasizes the importance of raising awareness and educating women about the potential consequences of delayed childbearing.

## Introduction

The most prevalent malignancy associated with pregnancy is breast cancer [1]. The prevalence of pregnancy-associated breast cancer has been estimated to be approximately 2% [1]. Pregnancy-associated breast cancer is defined as breast cancer diagnosed during pregnancy, breastfeeding, or up to 12 months after childbirth [2].

Childbirth at any age confers a transiently increased risk of breast cancer during the first decade postpartum [3]. A higher risk of breast cancer is associated with advanced maternal age during pregnancy [4], and as more women defer childbearing, as much their incidence of pregnancy-associated breast cancer is expected to increase [1, 4]. Women who childbirth at an advanced age experience a greater breast cancer risk peak in their postpartum [4], and in the same group of women, the cumulative risk of developing breast cancer remains elevated for many years and extends over two decades in women older than 30 years at first childbirth [3, 4]. In addition, pregnancy during, immediately before, or after a breast cancer diagnosis poses unique challenges due to the interaction between pregnancy hormones and breast cancer outcomes [5]. Moreover, breast cancer in young women (< 45 years) is associated with an increased risk of metastases and mortality [3].

This study aimed to compare a geographical location with a high incidence of delayed childbearing to a Country with a lower mean age at childbirth in order to investigate the overlap of women’s age distributions at childbearing and breast cancer diagnosis.

## Methods

### Study design and data sources

We conducted a retrospective analysis using a variety of data sources. We utilized information from the Surveillance, Epidemiology, and End Results (SEER) program, the United States National Center for Health Statistics as part of the National Vital Statistics System, the United Nations Population Division, the GLOBOCAN Cancer Observatory, the CLIO-INFRA project database, the Human Fertility Database, as well as anonymized local data. The SEER compiles cancer incidence and survival data from cancer registries covering approximately 34.6% of the U.S. population [6]. The National Vital Statistics System collects birth data from every state and territory in the United States [7]. We compared the breast cancer age-standardized rate (ASR) in the United States and Italy using GLOBOCAN data published by the International Agency for Research on Cancer (IARC) (GLOBOCAN Cancer Observatory—https://gco.iarc.fr/, accessed on 10th May 2023). Women’s age at childbearing was determined using data from the United Nations Population Division (https://population.un.org/dataportal, accessed on 10th May 2023). Data regarding women’s age at their first pregnancy was extracted by the Human Fertility Database (http://www.humanfertility.org, accessed on 10th May 2023). We obtained the life expectancy of women using data from the CLIO-INFRA project (http://www.clio-infra.eu/, accessed on 10th May 2023). The primary objective of the CLIO-INFRA project was to establish a data collection containing numerous socio-economic indicators and other environmental data to address the growing disparity between nations over time.

The research was carried out in accordance with the Helsinki Declaration and its subsequent amendments. Local data were used in accordance with local law as fully anonymized data or in accordance with a protocol previously approved by the Institutional Review Board. For the de-identified and publicly accessible data, neither institutional review board approval nor informed consent were required. We signed the data-use agreement and obtained authorization to access and utilize data from the SEER program. We adhered to this agreement while conducting this investigation.

### Study population

We included all women diagnosed with primary invasive breast cancer and reported to the SEER program (18 registers) between 2000 and 2018. We excluded individuals whose age and gender information was invalid or absent. We included all women who gave birth during the same time frame. All women diagnosed with breast cancer and women who gave birth at the academic hospital in Udine in the same time frame were included in the local database. Moreover, data from the United States and Italian populations were selected from the following databases: GLOBOCAN Cancer Observatory (U.S. data available from 1975 to 2016 and Italian data from 1988 to 2012), United Nations Population Division (data available from 1990 to 2022), the Human Fertility Database (U.S. data available from 1933 to 2011 and Italian data from 2004 to 2011), and the CLIO-INFRA project database (data available from 1890 to 2011).

### Considered variables and study outcome

We considered the incidence of female breast cancer, the average age of women at reproduction, and their average life expectancy at birth. In addition, we considered the age at childbearing, the age at breast cancer diagnosis, and the stage of breast cancer as variables. The primary outcome of interest was the distribution overlap of woman’s age at childbearing and their age at breast cancer diagnosis. As ASR, breast cancer incidence is presented. GLOBOCAN Cancer Observatory collected the ASR per 100,000 person-years based on the global standard population. The previously adjusted 5-year median age of childbearing was obtained from the United Nations Population Division database.

### Statistical analysis

Data elaboration and analysis were performed using R (version 4.3.0, 2023–04-21) [8]. Distribution areas were analyzed using the bayestestR package [9]. Random resampling was used to reduce samples greater than one million to one million items. In 1000 iterations, the consistency between the original and resampled cohorts was evaluated. Distributions of density were analyzed using the KernSmooth procedure. The age distributions were modeled for the data simulation as a normal distribution with the same mean and standard deviation. One million samples were generated at random. The area of overlap between women’s age distribution at childbearing and breast cancer diagnosis was evaluated by varying women’s mean age at childbearing between the mean values in the United States population sample and 35 years (the limit at which the average age of the local population tends to increase).

## Results

### Trends in breast cancer incidence and age at pregnancy

**Fig. 1:**
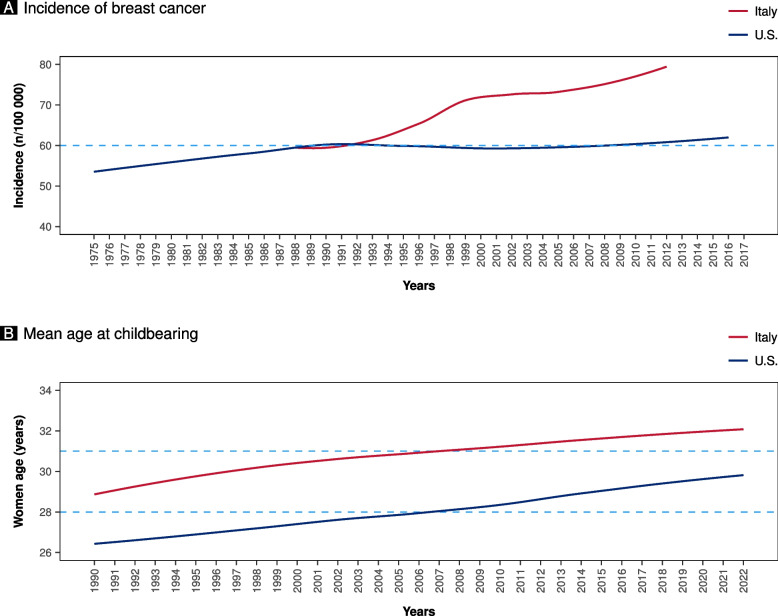
Panel **A** shows the incidence of breast cancer in Italy and the United States (U.S.) according to Globocan data (Globocan Cancer Observatory—https://gco.iarc.fr/). Panel **B** shows the mean age at childbearing (5-year median age at childbearing from United Nations data—https://population.un.org/dataportal)

**Fig. 2:**
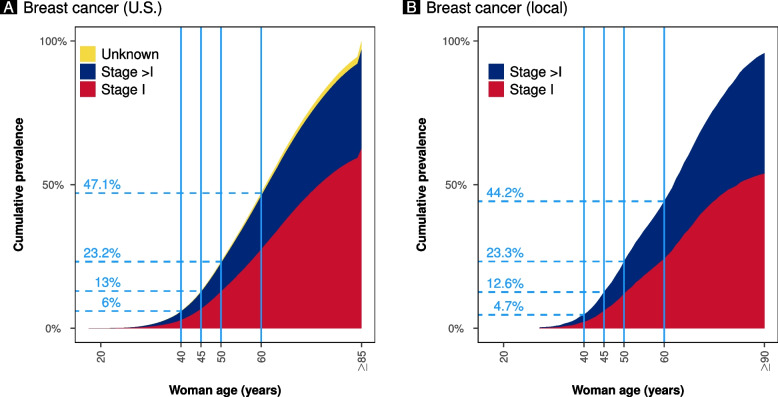
The plots show the cumulative prevalence of breast cancer according to women’s age at diagnosis. Panel **A** shows the United States (U.S.) data reported to the SEER program (18 registrations) between 2000 and 2018. Panel **B** shows the local data (2002–2018)

**Fig. 3:**
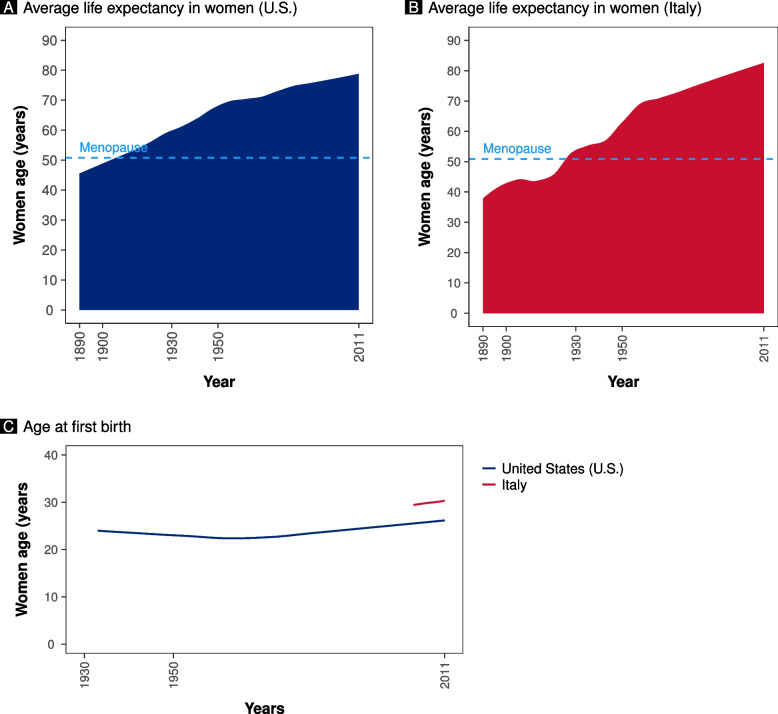
The graphs depict the demographic trends and menopause age in the United States (U.S.) and Italy. Panel **A** shows the female life expectancy at birth in the United States (blue area) (Clio Infra project, http://www.clio-infra.eu/) and the age at menopause [10]. Panel **B** shows the female life expectancy at birth in Italy (red area) (Clio Infra project, http://www.clio-infra.eu/) and the age at menopause [11]. Panel **C** shows the age at first birth (Human Fertility Database, http://www.humanfertility.org)

According to the Globocan data, the incidence of breast cancer has been increasing over time. This increment is particularly evident in Italy, starting from the late’90 s (Fig. 1A). Despite the apparent difference in breast cancer incidence between the United States and Italy, the cumulative prevalence according to women’s age is similar, with 23% of breast cancers diagnosed below 50 years of age (Fig. 2A and B). However, below 60 years of age, the prevalence was higher in the United States than in Italy (Fig. 2A and B). Despite a similar age at menopause, life expectancy has increased over time, reaching higher peaks in the Italian population than in the United States population (Fig. 3A and B) [10, 11]. According to the United Nations data, maternal age at pregnancy is steadily increasing in both countries, either considering age at pregnancy or age at first pregnancy (Figs. 1B and 3C).

### Age distributions at delivery and breast cancer diagnosis in local and U.S. populations

**Fig. 4:**
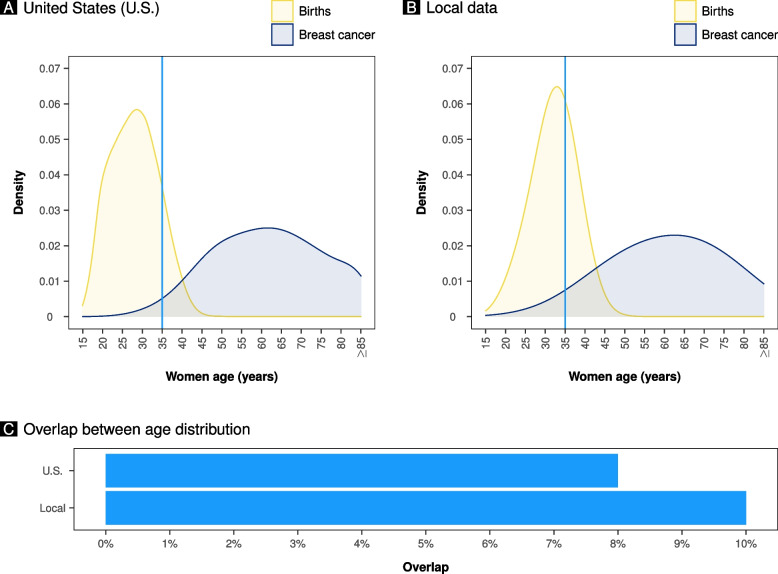
The density diagrams of age distribution at childbearing and breast cancer diagnosis, as well as the relative overlap area, are depicted in panels **A** and **B**. The data for the United States (U.S.) are shown in Panel **A** (National Vital Statistics System). Panel **B** displays local data. The overlap between age distribution at childbearing and breast cancer diagnosis in the United States and local data is depicted in panel **C**

Figure 4A shows the overlap between women’s age distribution at delivery and women’s age distribution at breast cancer diagnosis in the United States population. Meanwhile, Fig. 4B shows the overlap between women’s age distribution at delivery and women’s age distribution at breast cancer diagnosis in the local population. The overlap area was 9.92% in the local population and 7.78% in the United States population (Fig. 4C). The distribution of women age at delivery in the local population was overlapping at 70.15% with the United States cohort distribution. The mean age at delivery in the local population and in the U.S. cohort was respectively 32.12 (± 5.45) and 27.90 (± 6.06) years. The distribution of women age at breast cancer diagnosis in the local population was overlapping at 95.73% (only women aged less than 85 years) with the U.S. cohort distribution. The mean age at breast cancer diagnosis in the local population and in the U.S. cohort was respectively 61.89 (± 13.40) and 61.62 (± 13.54). Taking into consideration only women below 85 years of age, the mean values were respectively 60.87 (± 12.58) and 60.26 (± 12.66).

### Impact of increasing mean age at birth on the overlap between birth and breast cancer diagnosis age distributions

**Fig. 5:**
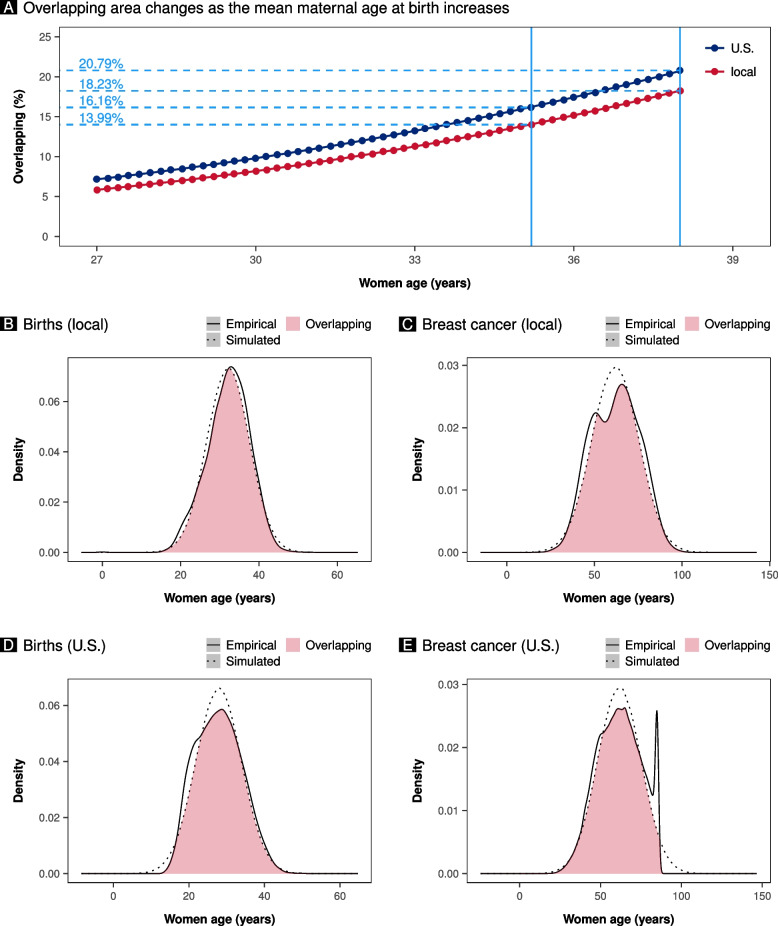
Panel **A** displays simulated data demonstrating how the overlapping area changes as the mean maternal age at birth in the United States (U.S.) and the local population increases. Panel **B** shows the overlap between the simulated and empirical age distribution of local births. Panel **C** shows the overlap between the simulated and empirical age distribution of local breast cancer diagnosis. Panel **D** shows the overlap between the simulated and empirical age distribution at birth of the United States (U.S.). Panel **C** shows the overlap between the simulated and empirical age distribution of the United States (U.S.) at breast cancer diagnosis. In each of panels **B**, **C**, **D**, and **E**, the overlap between simulated and empirical age distributions is consistently greater than 90 percent

We simulated the impact of an increase in the mean age at birth on the overlap between the age distributions at birth and breast cancer diagnosis. Figure 5A shows the simulated data in the local population and in the U.S. population. Supposing the local population had the same mean birth age as the U.S. population, there would be a 6.5% overlap between the age distribution at birth and at breast cancer diagnosis. If the U.S. population had the same mean birth age as the local population, the overlap between the age distributions at delivery and breast cancer diagnosis would be 10.17%. At a mean birth age of 35, the overlap between the age distribution at delivery and at breast cancer diagnosis would be 13.99% and 16.16% in the local and U.S. populations, respectively. If the average birth age was 38, the overlap between the age distribution at birth and at breast cancer diagnosis in the local and U.S. populations would be even 18.23% and 20.79%, respectively. As the mean birth age increases from 27 to 35 in both populations, the overlap between the distributions of birth age and age at breast cancer diagnosis increases by more than 200%.

In order to determine the consistency between the simulated and empirical distributions, we recalculated the data in Fig. 4C using the simulated distributions. For the same age at delivery as the empirical distributions, the overlap between the simulated and empirical ones was 10.30% for the local population and 7.88% for the U.S. population. In addition, the overlap between empirical and simulated distributions consistently exceeded 90% (Fig. 5B, C, D, and E).

## Discussion

With the progressive increase of women’s age at delivery, the overlap area between women’s age distribution at childbirth and at breast cancer diagnosis increased as well. In addition, while the average age at delivery increases from 27 to 35 years, the overlap between the distributions of age at delivery and at breast cancer diagnosis increases by more than 200%.

Breast cancer is the most prevalent cancer type among women worldwide [12], and pregnancy-associated breast cancer is the most prevalent malignancy during pregnancy and puerperium [3]. Then, the increasing convergence between age distributions at childbirth and at breast cancer diagnosis may be the result of the trend in delaying seeking pregnancy due to social and economical reasons, and may partially explain the observed increase in the number of breast cancers diagnosed during pregnancy [13]. Despite the overall low incidence of pregnancy-associated breast cancer, the increased overlap between the age distribution at pregnancy and at breast cancer diagnosis leads us to believe that the problem is expanding.

We observed a higher incidence of breast cancer in Italy than in the United States, which can be partially explained by the older age at first pregnancy among Italian women. However, there are additional explanations for this result. For instance, Italy has a longer life expectancy than the United States, and the longer one lives, the greater is her risk of developing breast cancer. This statement is supported by the fact that the average age of the local population is older than that of the United States. Furthermore, the introduction of population-based breast cancer screening in the 1990s, followed by its gradual implementation (started in the local region since 2005), surely improved the detection of early breast cancer and consequently increased the number of diagnoses although reduced overall cancer mortality [14–16]. This statement is consistent with the significant increase in the incidence of breast cancer since the 1990s. Thereafter, the observed higher incidence of breast cancer in the Italian population is most likely the result of multiple concomitant factors.

In recent years, delayed childbearing and increased age at first pregnancy have become a global issue [17–19]. Although the causes of delayed childbearing are multifactorial, it is essential to consider the long-term effects of this trend [19]. In particular, delayed childbearing may have substantial long-term effects on women’s health, and especially on breast cancer. For instance, parity status has been demonstrated to affect prognosis exclusively in the case of breast cancer [20], and increasing age at first pregnancy may increase the likelihood of a poor prognosis for breast cancer [3]. Moreover, the prognosis of young women affected by breast cancer (about 23% of all breast cancers) is reported to be negatively affected by the advanced age at first pregnancy [3, 20–24]. Thereafter, postpartum breast cancer represents a global health hazard that annually affects between 150,000 and 350,000 young mothers, putting them at a higher risk for metastasis and death [3].

Even though it is unclear why pregnancy and the age of first pregnancy have different effects on breast cancer, we can point out a few factors. Since the 1980s, it has been recognized that pregnancy can both inhibit and promote breast cancer [20, 25]. Before the age of 40, parous women experience a transient increase in breast cancer risk, whereas, after the age of 40, the same risk is increased in nulliparous women [20, 25]. The duration of increased breast cancer risk in parous women increases with advancing age at first pregnancy [21]. When the first pregnancy occurs after the age of 30, the increased risk of breast cancer can persist for more than 20 years [21]. This increased risk is validated by the biological mechanisms underlying mammary gland remodeling and atrophy [26, 27]. Mammary tissue expands approximately tenfold during pregnancy in preparation for lactation; mammary gland involution occurs when milk production ceases, either after delivery in the absence of breastfeeding or after weaning. Several observed mechanisms in the involuting gland may be responsible for metastasis. Inflammation, lymphangiogenesis, fibroblast activation, and deposition of collagen 1, fibronectin, and tenascinC-rich matrix are coordinated with the programmed death of the mammary epithelium [28–32]. All of these stromal characteristics resemble wound healing and contribute to cancer development. Significantly, the presence of a comparable pro-tumor breast involution program in the breast tissue of young, recently pregnant women provides a plausible link between breast involution and breast cancer outcome [30, 33]. In addition, there is a threefold increase in liver metastasis in postpartum breast cancer patients, but not in the lung, brain, or bone [34], likely due to a functional link between the mammary gland and liver established to sustain lactation, a condition in which the size of the liver doubles and its anabolic metabolism rises [34]. Thereafter, it undergoes the same involution as the mammary gland after lactation, which can promote metastasis. The increased prevalence of liver metastasis can also partially explain the increased mortality in this group of women since liver metastases are among the most fatal [35].

Furthermore, it has been demonstrated protracted breastfeeding to be protective against breast cancer. In contrast to maternal age or parity, research indicates that risk reduction is proportional to the cumulative duration of breastfeeding over the mother’s lifetime [3, 36]. This finding suggests that the longer a woman breastfeeds during her life, the greater her protection against breast cancer. This finding highlights the significance of encouraging and supporting women to breastfeed, not only for the health of their infants but also for their own long-term health benefits.

The relationship between neonatal and maternal morbidity and mortality and maternal age is represented by a U- or J-shaped curve, indicating that both very young and older maternal ages are associated with higher risks of adverse pregnancy outcomes [19, 37–41]. This evidence suggests that pregnancies of very young mothers (typically teenagers) and older mothers (often defined as 35 or older) are more likely to result in preterm birth, low birth weight, and higher mortality rates than those of intermediate-age range mothers [19, 37–41]. Adolescent and advanced maternal-age pregnancies pose significant public health risks, necessitating a multidisciplinary approach that combines healthcare, education, and policy interventions to support maternal and neonatal health. In addition to the inherent and immediate risks of pregnancy, when approaching a woman with a pregnancy at an advanced age, the potential implications for breast cancer risk must be discussed. One of these implications is that the woman’s age is one of the most influential risk factors for the development of breast cancer; at 35 years of age, the risk of developing breast cancer in the next 5 years is 1:333, at 40 years it is 1:167, at 45 it is 1:100, and at 50 years it is 1:77 [42, 43]. As a result, there is a hypothetical increased risk of being diagnosed with breast cancer during pregnancy or in the following period as one’s age increases. The second factor to consider is the increased risk of developing breast cancer in the subsequent years associated with the first pregnancy occurring at an advanced age. A first pregnancy after 30 may increase breast cancer risk for decades [21]. Many risk prediction models have used 30 years as the highest cut-off when assessing the risk of having the first child at an older age. However, this approach may underestimate the actual risk. As more data becomes available and the average age of delivery increases, we can expect to have more precise data to better define risk shades between 30 and 50 years of age.

The lack of consistent data on pregnancy-related breast cancer highlights the need for improved data collection methods to better understand this condition’s burden. This study’s findings have important implications for better understanding pregnancy-associated breast cancer and cancer in young women, as well as developing more effective breast cancer screening strategies. For example, mammary gland ultrasound examinations in women over the age of 35 may aid in the early detection of breast cancer during pregnancy and after delivery. Clinicians should be aware and consider earlier and more frequent breast cancer screening for women who postpone childbearing. Recognizing the risk is the first step in designing clinical trials to determine the cost-effectiveness of implementing new screening modalities. The scarcity of comprehensive data emphasizes the critical need for clinical studies specifically designed to assess the efficacy and benefits of targeted screening methods for pregnancy-associated breast cancer and breast cancer in young women, such as mammary gland ultrasound examinations. Such studies are necessary to create evidence-based guidelines that can be used to guide clinical practice, ensuring that screening strategies are both scientifically relevant and optimized for early detection. Conducting rigorous clinical research in this area will not only validate the proposed screening approaches but may also result in the development of new screening protocols tailored to the specific risks faced by pregnant women of advanced maternal age. By implementing these strategies, we may be able to improve early detection of pregnancy-associated breast cancer and young women’s breast cancer, potentially improving their prognosis.

Several limitations of the present investigation must be acknowledged. First, reliance on retrospective datasets or retrospective and public datasets from various sources introduces inherent limitations associated with data quality and precision. Despite efforts to ensure data integrity and validity, datasets may contain inconsistencies and biases due to variations in data collection methodologies, reporting practices, and data quality controls among the original sources. These variations may affect the generalizability of the results. Secondly, the scope of the study is limited by the specific datasets included, which may not capture the entirety of pertinent information or the complete range of variables required for a comprehensive analysis. Our research focused on the age at which breast cancer is diagnosed and the age at which pregnancy occurs. However, it is essential to note that other factors can raise the risk of breast cancer. These factors include early menarche age, alcohol consumption, obesity, or physical inactivity [44–47]. Some of these factors, such as early menarche age and obesity, are on the rise, potentially contributing to the increasing incidence of breast cancer [47–49]. These factors, however, are unlikely to have a noteworthy impact on the association between advanced pregnancy age and the risk of breast cancer; instead, they may contribute to the risk, as highlighted in the predictive models also used in clinical practice [42].

Moreover, it should be noted that approximately 10% of breast cancers are triple negative, and an additional 10% express simultaneously hormone receptors and Her2. Younger women and Black American women are more likely to develop these neoplasms. Although this is an essential factor to consider when personalizing risk, we believe it should not have an impact on the analyses performed in this study because the majority of women included in the SEER dataset are non-Hispanic White, and the general age distribution overlaps with the age distribution of non-Hispanic White women by 96%. Considering this, it should be underlined that the specific characteristics and context of the datasets used may limit the generalizability of the study’s findings to other populations or contexts. The datasets included in this analysis may represent a particular time period, geographic region, or demographic group, limiting the external validity of the study’s findings. Despite these limitations, the study’s findings’ generalizability has been enhanced by including nationwide data and data from two countries.

Reflecting on the insightful observations made throughout this paper, it is clear that there is significant scope for further research into pregnancy-related cancers and the effects of advanced age at first pregnancy on future breast cancer risk. This study emphasizes the importance of further research into the complexities and nuances of cancers that occur during pregnancy, as well as the risk factors associated with advanced maternal age at first pregnancy. First, more precise epidemiology on the link between pregnancy and cancer is required, as is more structured data collection in the appropriate registers. Furthermore, future research should broaden our understanding by investigating various factors that may influence pregnancy-associated cancers, such as genetic predispositions, lifestyle influences, and healthcare practices. Furthermore, longitudinal studies that track women’s long-term health behind pregnancy could provide invaluable information for developing more effective screening, prevention, and treatment strategies.

## Conclusions

In conclusion, our analysis highlights the considerable overlap between the age distributions at childbirth and at breast cancer diagnosis, especially as the average age at first pregnancy continues to rise. Our findings indicate that this overlap increases dramatically between the ages of 27 and 35, emphasizing the importance of raising awareness and education about the timing of childbearing and the importance of breast cancer screening. These findings highlight the importance of providing comprehensive information and support to women in order for them to make more informed decisions about their reproductive options and cancer prevention strategies. By encouraging informed decision-making, we hope to improve women’s reproductive health and contribute to the early detection of breast cancer, potentially improving outcomes for women of all ages.

## Data Availability

Local data that support the findings of this study are available. However, restrictions apply to the availability of these data, which were used under license for the current study and are not publicly available. Data are, however, available from the authors upon reasonable request and with permission of the Internal Review Board. All other data were extracted from published de-identified or publicly available datasets; thus, they are publicly available: https://gco.iarc.fr/ (GLOBOCAN Cancer Observatory); https://www.humanfertility.org/Data/ZippedDataFiles (Human Fertility Database); http://www.clio-infra.eu/ (CLIO-INFRA project); https://seer.cancer.gov/ (Surveillance, Epidemiology, and End Results); https://population.un.org/dataportal (United Nations Population Division); and https://www.cdc.gov/nchs/data_access/vitalstatsonline.htm (US National Center for Health Statistics as part of the National Vital Statistics System).

## Abbreviations

ASR: Age-standardized rate
IARC: International Agency for Research on Cancer
SEER: Surveillance, Epidemiology, and End Results
U.S.: United States
